# Donor mesenchymal stem cell-derived neural-like cells transdifferentiate into myelin-forming cells and promote axon regeneration in rat spinal cord transection

**DOI:** 10.1186/s13287-015-0100-7

**Published:** 2015-05-27

**Authors:** Xue-Cheng Qiu, Hui Jin, Rong-Yi Zhang, Ying Ding, Xiang Zeng, Bi-Qin Lai, Eng-Ang Ling, Jin-Lang Wu, Yuan-Shan Zeng

**Affiliations:** Key Laboratory for Stem Cells and Tissue Engineering (Sun Yat-sen University), Ministry of Education, Guangzhou, 510080 China; Department of Histology and Embryology, Zhongshan School of Medicine, Sun Yat-sen University, Guangzhou, 510080 China; Institute of Spinal Cord Injury, Sun Yat-sen University, Guangzhou, 510120 China; Co-innovation Center of Neuroregeneration, Nantong University, Nantong, 226001 China; Department of Electron Microscope, Zhongshan School of Medicine, Sun Yat-sen University, Guangzhou, 510080 China; Department of Anatomy, Yong Loo Lin School of Medicine, National University of Singapore, Singapore, 117597 Singapore

## Abstract

**Introduction:**

Severe spinal cord injury often causes temporary or permanent damages in strength, sensation, or autonomic functions below the site of the injury. So far, there is still no effective treatment for spinal cord injury. Mesenchymal stem cells (MSCs) have been used to repair injured spinal cord as an effective strategy. However, the low neural differentiation frequency of MSCs has limited its application. The present study attempted to explore whether the grafted MSC-derived neural-like cells in a gelatin sponge (GS) scaffold could maintain neural features or transdifferentiate into myelin-forming cells in the transected spinal cord.

**Methods:**

We constructed an engineered tissue by co-seeding of MSCs with genetically enhanced expression of neurotrophin-3 (NT-3) and its high-affinity receptor tropomyosin receptor kinase C (TrkC) separately into a three-dimensional GS scaffold to promote the MSCs differentiating into neural-like cells and transplanted it into the gap of a completely transected rat spinal cord. The rats received extensive post-operation care, including cyclosporin A administrated once daily for 2 months.

**Results:**

MSCs modified genetically could differentiate into neural-like cells in the MN + MT (NT-3-MSCs + TrKC-MSCs) group 14 days after culture in the GS scaffold. However, after the MSC-derived neural-like cells were transplanted into the injury site of spinal cord, some of them appeared to lose the neural phenotypes and instead transdifferentiated into myelin-forming cells at 8 weeks. In the latter, the MSC-derived myelin-forming cells established myelin sheaths associated with the host regenerating axons. And the injured host neurons were rescued, and axon regeneration was induced by grafted MSCs modified genetically. In addition, the cortical motor evoked potential and hindlimb locomotion were significantly ameliorated in the rat spinal cord transected in the MN + MT group compared with the GS and MSC groups.

**Conclusion:**

Grafted MSC-derived neural-like cells in the GS scaffold can transdifferentiate into myelin-forming cells in the completely transected rat spinal cord.

**Electronic supplementary material:**

The online version of this article (doi:10.1186/s13287-015-0100-7) contains supplementary material, which is available to authorized users.

## Introduction

Severe spinal cord injury (SCI) results in complete or partial loss (or both) of motor and sensory function below the level of the lesion, and this loss is attributed to loss of cells, nerve fiber tract disruption, and demyelination through the primary mechanical insult and the secondary reactive damage such as inflammation, oxidative stress, excitotoxicity, and increase in free radicals [1–4]. Owing to its complicated pathophysiology, there is no effective treatment for SCI so far [2, 5]. Recent studies have shown that endogenous nervous tissue stem cells activate, proliferate, and migrate after SCI [6, 7], and this may open a new therapeutic avenue based on stem cells. However, endogenous stem cells are limited to rehabilitate motor and sensory function [8]. With the development of regenerative medicine, tissue-engineered exogenous stem cell transplantation has become a promising strategy to restore the structure and function of injured spinal cord [9].

Mesenchymal stem cells (MSCs), as important seed cells of tissue engineering, have received the most attention for treatment of central nervous system injury in view of their ease of culturing and low immunogenicity, immunoregulation, pro-survival, and neurogenic differentiation properties [10, 11]. Indeed, the capability of transdifferentiation of MSCs into neurons and myelinating cells ex vivo and in vivo studies [12–17] has made them a stem cell of choice among others in SCI rehabilitation. Neurotrophic factors (NTFs), a family of proteins, promote the survival and growth of developing neurons and maintain the function of mature neurons [18]. It has also been reported that NTFs prevent neuron death and promote axon regrowth in SCI [19–21] and induce adult stem cell differentiation [22–25]. In our previous studies, we reported that neurotrophin-3 (NT-3)/TrkC signal pathway promotes MSC differentiation. This was strongly evidenced by the fact that Schwan cells (SCs) modified by NT-3 gene could induce MSCs overexpressing NT-3 receptor-TrkC to differentiate into neural cells in two-dimensional (2D) and three-dimensional (3D) culture in vitro [26, 27]. However, the low neural differentiation frequency of MSCs in the 2D induction has limited its application. Given that cells in a 3D environment in vitro would closely mimic cells in vivo and furthermore that they present predominant properties compared with those in a 2D environment, such as metabolism [28, 29], gene expression and protein synthesis [30, 31], proliferation [32], and differentiation [27, 33], the 3D gelatin sponge (GS) scaffold was constructed and adopted to support the growth and neural differentiation of MSCs [34].

To enhance TrkC overexpressing MSCs differentiating into neural cells effectively, NT-3 and compatible 3D material are essential. Moreover, an available vector that could maintain expression of NT-3 has become an important yet unresolved issue. Another consideration would be the use of SCs which are known to secrete various NTFs, such as nerve growth factor, ciliary neurotrophic factor, brain-derived neurotrophic factor, fibroblast growth factor, and NT-3 [35]. But these may affect and complicate the effect of NT-3/TrkC interaction on MSC transdifferentiation. In addition, grafted SCs can take part in myelination of regenerating axon [36]. Therefore, if MSCs were to be co-transplanted with SCs together, the above-mentioned factors of SCs may interfere with the mechanism and accurate evaluation of MSCs repairing injured spinal cord. Therefore, only MSCs were used in this study as an effective delivery vehicle overexpressing NT-3 or TrkC. Separately, MSC transdifferentiation into different neural phenotypes in vitro and in vivo has been reviewed by Maltman et al. [37], who indicated that the complex niche may educate the MSCs to transdifferentiate into different neural phenotypes. The present study attempted to explore whether NT-3 gene-modified MSCs could promote TrkC gene-modified MSCs to differentiate into neural-like cells in a GS scaffold and, more importantly, whether the grafted MSC-derived neural-like cells could maintain neural features or transdifferentiate into other neural cells such as myelin-forming cells in the completely transected rat spinal cord.

## Methods

### Cell culture

MSCs were isolated as previously described from Sprague-Dawley (SD) rats (Sun Yat-sen University, Guangzhou, China, for in vitro experiments) or green fluorescent protein (GFP) transgenic SD rats (Osaka University, Osaka, Japan) which ubiquitously express enhanced GFP under the control of the β-actin promoter (for in vivo experiments) [26]. Because MSCs of younger rats have a stronger differentiation potential [38–40], about 1-week-old SD rats were sacrificed. The femurs and tibiae were removed, cleaned of all connective tissue, and clipped to expose the marrow. The bone marrow was flushed and cultured in low-glucose Dulbecco’s modified Eagle’s medium (L-DMEM) (Gibco, a brand of Thermo Fisher Scientific, Waltham, MA, USA) supplemented with 10 % fetal bovine serum (FBS) (TBD Co., Tianjin, China) and 4 mM L-glutamine (Invitrogen, a brand of Thermo Fisher Scientific) in an incubator with 5 % CO_2_ at 37 °C. When the cells reached about 80 % confluency, the MSCs were passaged. Cells used in the present study were in passage 3–5.

### Gene overexpressing and seeding of gene-modified MSCs in the GS scaffold

The MSCs were engineered to overexpress TrkC or NT-3 by recombinant adenovirus containing TrkC gene (Ad-TrkC) or NT-3 gene (Ad-NT-3), respectively. In accordance with procedures described in our previous studies, MSCs were transfected by Ad-TrkC—300 multiplicity of infection (MOI)—for 3 hours [26]. Then the medium was replaced by DMEM with 10 % FBS to allow growth for another 24 hours. Another set of MSCs was transfected by Ad-NT-3 (200 MOI) the same way.

Three-dimensional GS (3D GS) scaffolds were prepared as previously described [34]. The scaffolds were divided into four groups: the MSCs (M), NT-3-MSCs (MN), TrkC-MSCs (MT), and NT-3-MSCs + TrkC-MSCs (MN + MT, mixed in a 1:1 ratio) groups. In total, 5 × 10^5^ cells in 10 μl of culture medium were seeded to each scaffold. Then the scaffolds continued to incubate for 14 days in DMEM with 5 % FBS, and the culture medium was replaced every 2 days.

### Surgical procedure and cell transplantation

Adult female SD rats (220–250 g, supplied by the Experimental Animal Center of Sun Yat-sen University) were used in this study. Animals were given cyclosporin A (10 mg/kg per rat) subcutaneous injection in the belly 3 days before surgery. The animals were anesthetized with 1 % pentobarbital sodium (40 mg/kg by intraperitoneal injection) to minimize suffering. After laminectomy at the T9 vertebral level, the spinal cord was transected and a 2-mm cord segment, including visible spinal roots, was completely removed at the T10 spinal cord level. Then MN + MT scaffold (the MN + MT group), M scaffold (the MSC group), or gelatin sponge scaffolds (the GS group) after 14 days of culture were transplanted to fill up the injury gap of spinal cord, respectively. After the surgical procedure, the overlying musculature and skin were sutured sequentially. The rats received extensive post-operation care, including intramuscular injection of penicillin (50,000 U/kg per day) for 3 days and manual emiction three times daily until their automatic micturition function was re-established. Cyclosporin A was administrated once daily for 2 months. All experimental protocols and animal handling procedures were approved by the Animal Care and Use Committee of Sun Yat-sen University and were consistent with the National Institutes of Health Guide for the Care and Use of Laboratory Animals.

### Tissue processing

All rats were sacrificed at the end of 3 days or 8 weeks after the scaffolds were transplanted. The rats were deeply anesthetized with 1 % pentobarbital sodium (50 mg/kg by intraperitoneal injection). Afterward, they (except those that were used in the immune-electron microscopic observations) were transcardially perfused with 100 ml of normal saline containing 0.002 % NaNO_2_ and heparin, followed by a fixative containing 4 % paraformaldehyde in 0.1 M phosphate buffer (PB) (pH 7.4). The spinal cord tissue containing the injury/graft site was dissected, post-fixed in the fixative overnight, and transferred into 30 % sucrose in 0.1 M PB at 4 °C until the tissue sunk. The T8-T12 successive segments of the spinal cord were cut into longitudinal sections (thickness of 25 μm) which were mounted on gelatin-coated slides for immunofluorescence staining.

### Immunofluorescence staining

Specific proteins were detected by using immunofluorescence staining (IFS) as reported previously [41]. Briefly, the post-fixed spinal cord was sectioned at 20-μm thickness with a cryostat. The sections were rinsed with 0.01 M phosphate-buffered saline (PBS) three times, blocked with 10 % goat serum for 30 min, and incubated with primary antibodies mixed in 0.3 % Triton X-100 overnight at 4 °C. After rinsing in PBS, the sections were incubated with secondary antibodies and examined under a fluorescence microscope. A summary of antibodies used is as follows: rabbit anti-β-tubulin III (Tju-1) (1:300, Sigma-Aldrich, St. Louis, MO, USA), mouse anti-microtubule-associated protein 2 (Map2) (1:1000, Sigma-Aldrich), mouse anti-glial fibrillary acidic protein (GFAP) (1:300, Sigma-Aldrich), mouse anti-neurofilament 200 (NF200) (1:300, Sigma-Aldrich), rabbit anti-myelin basic protein (MBP) (1:600, Chemicon, now part of Millipore Corporation, Billerica, MA, USA), and mouse anti-adenomatous polyposis coli (APC) (1:200, Abcam, Cambridge, UK).

### Western blotting

Five animals in each group were sacrificed 8 weeks after the surgery for Western blotting (WB) analysis. The injury site of the spinal cord, including the whole injury/graft site (about 2 mm) and excluding as much as possible the uninjury tissue, was dissected and homogenized in ice-cold whole cell lysis buffer (Lysis buffer, Boster, Wuhan, China) containing 4 % protease inhibitor cocktails (Cocktail, Roche, Basel, Switzerland) for 10 min. Then homogenate was centrifuged (13,200 revolutions per minute, 20 min, 4 °C), and the supernatant was collected. After protein concentration was assayed by using a bicinchoninic acid assay (BCA) protein assay in accordance with the instructions of the manufacturer (BCA protein assay, Thermo Fisher Scientific), 20 μg of total protein from each sample was electrophoresed across 10 % sodium dodecyl sulfate-polyacrylamide gel (90 min at 100 V). The protein was then transferred from the gel to a polyvinylidene fluoride membrane (Millipore Corporation) by using a wet transfer method (180 min at 300 mA). Then the membranes were blocked in skim milk (5 %, 1 h) before incubation with primary antibodies: rabbit anti-NF200 (1:2000, Sigma-Aldrich), mouse anti-chondroitin sulfate proteoglycans clone CS-56 (CSPG) (1:1000, Sigma-Aldrich), mouse anti-GFAP (1:2000, Sigma-Aldrich), goat anti-MBP (1:1000, Santa Cruz Biotechnology, Inc., Dallas, TX, USA), and mouse anti-β-actin (1:5000, Sigma-Aldrich). The membranes were washed three times with TBST (0.5 % Tween 20 in Tris-buffered saline), incubated with horseradish peroxidase (HRP)-conjugated secondary antibodies (goat anti-rabbit HRP-conjugated antibody, 1:5000, 2 h and goat anti-mouse HRP-conjugated antibody, 1:5000, 2 h, Cell Signaling Technology, Beverly, MA, USA; horse anti-goat HRP-conjugated antibody, 1:5000, 2 h, Jackson ImmunoResearch Laboratories, Inc., West Grove, PA, USA), and visualized by using an ECL (enhanced chemiluminescence) Western Blot Kit (Kangwei, Beijing, China). Integrated intensities of each target protein were analysed by ImageJ and normalized against the β-actin loading control for each sample.

### Ultrastructural observations

After 14 days of culture, histological appearance of MSC-derived neuron-like cells in the scaffolds was examined under a scanning electron microscope (SEM). For SEM examination, scaffolds were firstly washed three times with PBS, fixed in 2.5 % glutaraldehyde for 90 min, dehydrated with a series of graded ethanol, and freeze-dried for 2 days. The dried samples were coated with gold and examined under the SEM (Philips XL30 FEG, Philips, Eindhoven, The Netherlands).

The scaffolds with cells were also examined under a transmission electron microscope (TEM) as previously described [36]. Scaffolds with seeded cells were fixed with 2.5 % glutaraldehyde at 4 °C for 1 h, post-fixed with 1 % osmic acid for 1 h, dehydrated through graded ethanol, embedded in Epon overnight, and followed by polymerization at 60 °C for 48 h. Ultrathin sections were cut with an ultramicrotome (Reichert E, Co., Vienna, Austria) and examined under the TEM (Philips CM 10).

For immune-electron microscope (IEM) detection, the anesthetized rats were transcardiacally perfused with physiological saline containing 187.5 U/100 ml heparin and followed by 0.1 M PB (pH7.4) containing 4 % paraformaldehyde, 0.1 % glutaraldehyde, and 15 % saturated picric acid. The area of spinal cord of interest was dissected, post-fixed overnight at 4 °C with fresh fixative, and cut into 50-μm coronal sections by vibratome. The sections were transferred into cryoprotectant solution (25 % sucrose and 10 % glycerol in 0.1 M PBS) overnight at 4 °C. Next, a quick freeze-thaw was performed three times by liquid nitrogen. This was followed by washing three times with PBS. The sections were then blocked by 20 % goat serum for 1 h and incubated with primary antibody (mouse anti-GFP, Millipore Corporation) containing 2 % goat serum for 24 h at 4 °C. After rinsing with PBS, immunohistochemical staining was performed by Vectastain ABC-HRP Kits (Vector Laboratories, Burlingame, CA, USA). The GFP in grafted cells was localized by 3, 3′-diaminobenzidine (DAB) substrate. The sections were then dehydrated in a graded series of ethanol and embedded in Epon 812 for ultrathin sectioning. After staining with uranyl acetate, the sections were examined under the TEM.

### Electrophysiology

Eight weeks after the spinal cord surgery, motor evoked potentials (MEPs) of the animals (*n* = 5 for each group, except *n* = 3 for the normal group) were recorded to assess motor nerve conduction by BL-420E Data Acquisition Analysis System (Taimeng, Chengdou, China) as previously described [42]. Briefly, after general anesthesia (ketamine 40 mg/kg and 1 % sodium pentobarbital 30 mg/kg), the sensorimotor cortex (SMC) and sciatic nerve were exposed. The stimulating electrode and recording electrode were connected to the SMC and sciatic nerve, respectively. MEPs were elicited by electrical stimulation of the SMC, located 2 mm lateral to the midline and 2 mm posterior to the bregma. Single pulse stimulation of 50-ms duration was employed. A voltage is adjusted to produce the maximum amplitude of MEPs. Normally, it is appropriate to adjust the voltage to 6–10 V. After this, the amplitude and latency of MEPs were obtained.

### Morphological quantification

For in vitro quantification of immunopositive cells, 10 separate sections from each scaffold were selected (*n* = 5 in each group). After IFS with respective antibodies, five fields (four corners and one center) at 200× magnification for each of the sections were counted. The percentage of immunopositive cells was obtained by the total number of immunopositive and Hoechst33342 double-positive cells divided by the total number of all Hoechst33342-positive cells.

For the quantification of axonal regeneration in vivo, the NF200-positive axons were quantified by using ImageJ analysis of immunofluorescence-labeled sections and following the procedure described earlier [43]. Briefly, two random fields within the rostral, central, and caudal areas of the injury/graft site of spinal cord were respectively measured in a longitudinal section (every ninth section of each animal; total of 10 sections per rat; *n* = 5 for each group). The pixel number occupied by immunofluorescence-labeled axons at 200× magnification was measured in sections and normalized by total pixels of one field (2088 × 1550 pixels) to obtain mean axon density per field, respectively, in the rostral, central, and caudal areas of the injury/graft site. Thresholding values on stained images were chosen to make sure that only immunolabeled axons were included. Weak non-specific background labeling was undetected.

For analysis of the survival of injured host neurons in vivo, transverse sections from two lumbar segments (L1 and L3) of spinal cord were stained with neutral red. The number of neurons—Clarke’s nucleus (CN) and ventral horn—was counted in every ninth section of L1 and L3 segments per animal. Only neurons with well-delineated nuclear outline in L1 CN and L3 ventral horn of spinal cord were counted under the light microscope. The data were presented as neuron per section. Neuron calculation in the present study was performed in a blinded fashion.

### Behavior assessments

All animals were gently handled and acclimatized to an open field for the locomotor test once daily for seven sessions before surgery. Also, they were trained to climb onto a 45° inclined grid once daily for 7 days. Each animal had to completely pass their bodies and both forelimbs and hindlimbs to the top of the grid to succeed in the performance three times each session. After cell transplantation or control treatment, the Basso, Beattie, and Bresnahan (BBB) open-field locomotor rating scale was performed every week for up to 8 weeks [44]. During open-field locomotion testing, SCI rats were allowed to explore freely for 5 min to assess hindlimb locomotor frequency, joint movement range, and coordination. In the final week, the 45° inclined grid climbing test was performed according to what was reported previously by Ramon-Cueto et al. [45].

### Statistical analysis

Data are expressed as the mean ± standard deviation. All statistical analyses were performed by using SPSS 17.0 statistical software (IBM Corporation, Armonk, NY, USA). The data were analyzed by using one-way analysis of variance. Post hoc least significant difference test for comparative pairs of groups was used. The significance level was set at a *P* value of less than 0.05.

## Results

### Expression of TrkC gene and NT-3 gene in mesenchymal stem cells

**Fig. 1:**
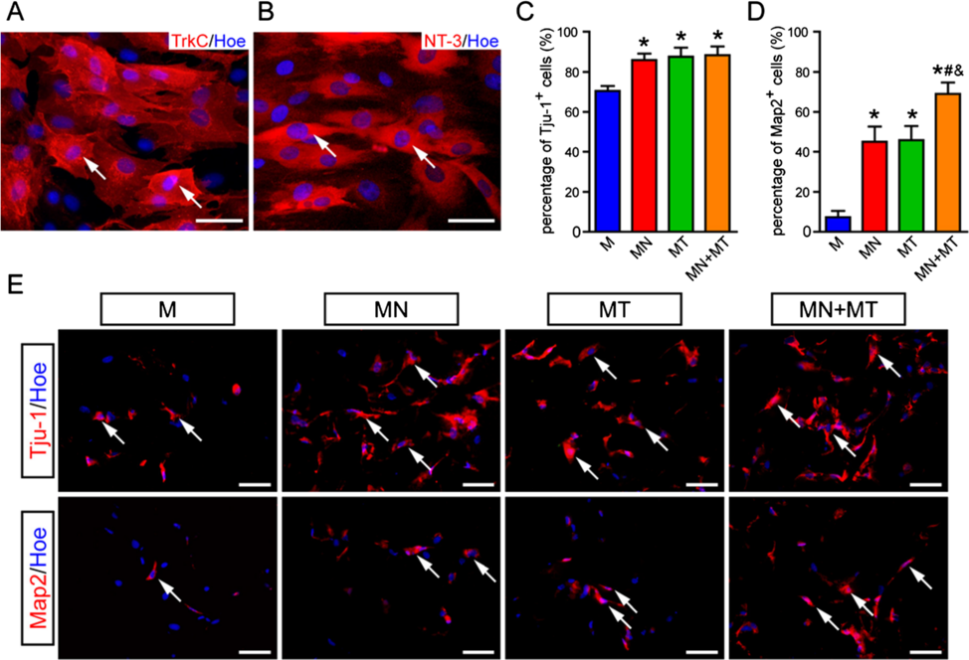
Identification of TrkC or NT-3 gene transfection and neural differentiation of co-cultured TrkC-MSCs and NT-3-MSCs in the 3D GS scaffold. **a**, **b** MSCs at 2 days after infected with Ad-TrkC and Ad-NT-3 in vitro, respectively. Note that TrkC-positive (**a**) and NT-3-positive (**b**) MSCs are evident. **c**, **d** Bar charts show the percentage of Tju-1-positive (**c**) and Map2-positive (**d**) cells in the M, MN, MT, and MN + MT groups. Asterisk indicates statistical significance compared with the M group (*P < 0.05), pound sign indicates significance compared with the MN group (^*#*^ *P* < 0.05), and ampersand indicates significance compared with the MT group (^*&*^ *P* < 0.05). The data were presented as mean ± standard deviation (*n* = 5), and one-way analysis of variance with least significant difference test statistics was performed. **e** Cells in the 3D GS scaffold were immunostained by Tju-1 and Map2 antibodies 14 days after culture. Tju-1-positive and Map2-positive cells were observed in all groups. Scale bars = 20 μm. 3D, Three-dimensional; GS, Gelatin sponge; M, MSCs; Map2, Microtubule-associated protein 2; MN, NT-3-MSCs; MSCs, Mesenchymal stem cells; MT, TrkC-MSCs; NT-3, Neurotrophin-3; Tju-1, β-tubulin III; TrkC, Tropomyosin receptor kinase C

To examine the protein expression of TrkC and NT-3 in genetically modified MSCs before seeding into GS scaffolds, IFS was used. About 75 % of TrkC-MSCs had TrkC-positive staining (Fig. 1a), and about 70 % of NT-3-MSCs had NT-3-positive staining (Fig. 1b).

### Neural marker expression of the mesenchymal stem cells in GS scaffold

To investigate whether NT-3-MSCs could promote TrkC-MSCs differentiating into neural-like cells in GS scaffold after 14 days of culture in vitro, the immature neuron marker Tju-1, mature neuron marker Map2, astrocyte marker GFAP, and oligodendrocyte marker APC were used to detect the differentiating MSCs by IFS. The results showed that about 88.25 ± 4.31 % of the MSCs expressed Tju-1 in the MN + MT group, which was significantly higher (*P* < 0.05) than the M group (70.43 ± 2.55 %); however, there was no obvious difference (*P* > 0.05) compared with the MN (85.88 ± 3.13 %) and MT (87.54 ± 4.47 %) groups (Fig. 1c, e). The percentage of Map2-positive cells was statistically different (*P* < 0.05) between the MN + MT group (68.90 ± 5.70 %) and other groups (the M group: 7.36 ± 3.12 %; the MN group: 44.98 ± 7.68 %; the MT group: 45.78 ± 7.11 %) (Fig. 1d, e). GFAP- and APC-positive cells (data not shown) were not observed in all groups.

### Morphological feature and synaptogenesis of the mesenchymal stem cells in vitro

**Fig. 2:**
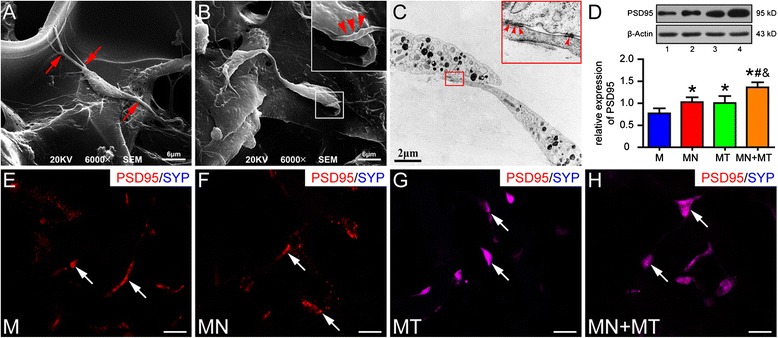
Detection of mesenchymal stem cell (MSC)-derived neuron-like cells in gelatin sponge (GS) scaffold 14 days after culture. **a**, **b** Scanning electron microscopy (SEM) shows differentiating MSCs bearing long and branched processes (**a**) (red arrows) on the surface of GS. One of the processes appears to make contact with another cell body (**b**) (red arrowheads). **c** Transmission electron microscopy shows a few synapse-like structures between two processes of differentiating MSCs (red arrowheads). **d** PSD95 expression was detected by Western blotting (1 = the M group, 2 = the MN group, 3 = the MT group, 4 = the MN + MT group). PSD95 level is highest in the MN + MT group compared with the M, MN, and MT groups (One-way analysis of variance with least significant difference test statistics was performed; *P < 0.05, ^*#*^ *P* < 0.05, ^*&*^ *P* < 0.05). **e**–**h** Expression of PSD95 and SYP was detected by immunofluorescence staining in the M, MN, MT, and MN + MT groups. All groups exhibited PSD95-positive cells, whereas SYP-positive cells were absent in the M and MN groups (white arrows). Scale bars = 20 μm in **(**e–h). M, MSCs; MN, NT-3-MSCs; MT, TrkC-MSCs

At 14 days after culture, genetically modified MSCs in GS scaffold in the MN + MT group were observed under the SEM. The differentiating MSCs were spindle-shaped cells bearing long branched processes and showed a good adherence on the GS surface (Fig. 2a). Some synapse-like contacts were observed (Fig. 2b). By TEM, synapse-like structures between cell processes were observed. The contact sites exhibited presynaptic electron-dense, a synaptic cleft, and a distinct post-synaptic electron-dense in the MN + MT group (Fig. 2c). By WB analysis, PSD95 expression level was higher in the MN + MT group than in other groups (Fig. 2d; *P* < 0.05). Moreover, PSD95 and SYP co-expressing cells were found with IFS only in the MN + MT and MT groups (Fig. 2e-h). The results suggest that genetically modified MSCs exhibit a neuron-like cell phenotype, such as the occurrence of synapse-like structures and expression of synaptic protein in GS scaffold 14 days after culture.

### Survival and differentiation of grafted mesenchymal stem cells modified genetically in vivo

**Fig. 3:**
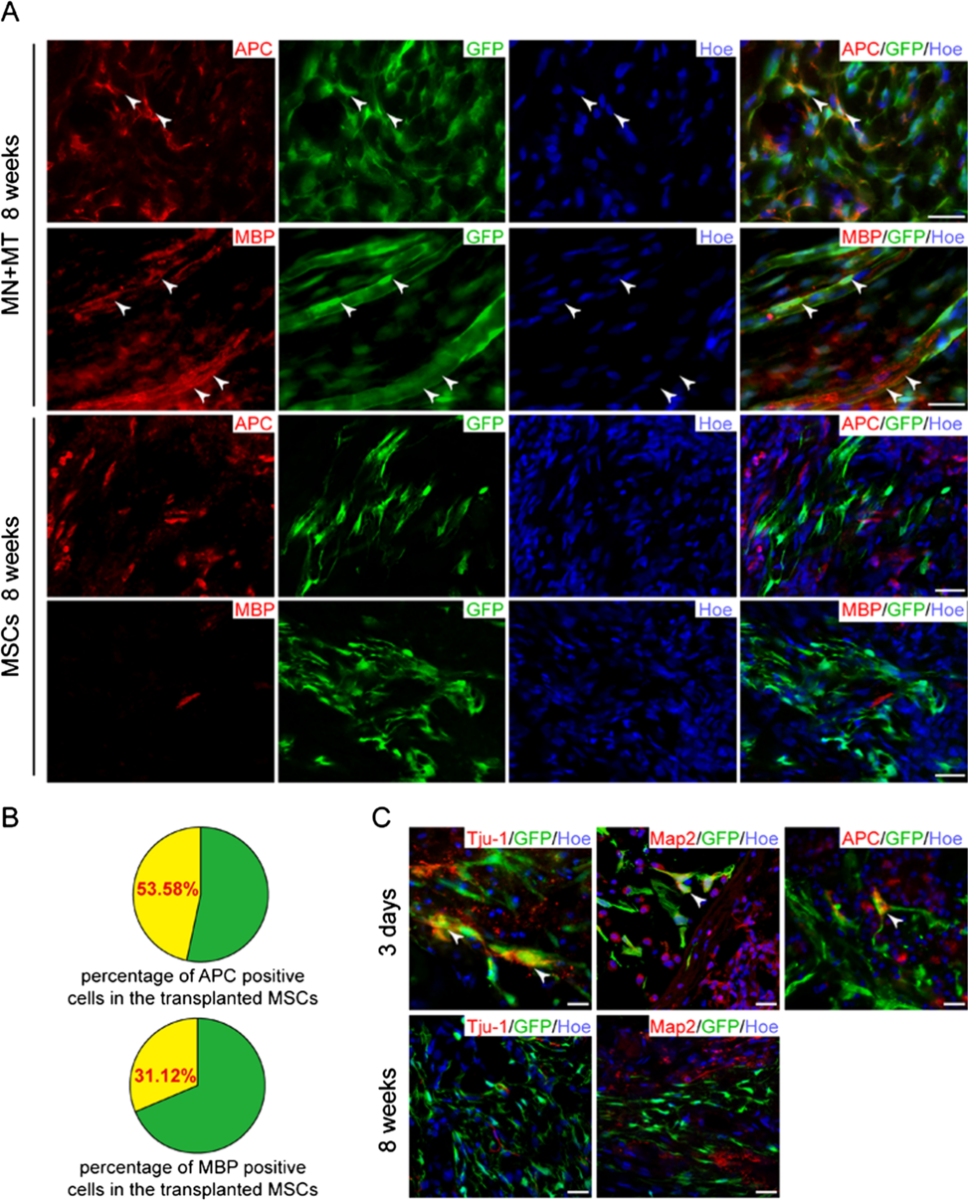
Transdifferentiation of transplanted mesenchymal stem cell (MSC)-derived neural-like cells in the injury/graft site of spinal cord. **a** Some of the grafted MSCs transdifferentiated into myelin-forming cells (white arrowheads, adenomatous polyposis coli (APC)-positive and myelin basic protein (MBP)-positive) in the NT-3-MSC (MN) + TrkC-MSCs (MT) group, but not in the MSCs group, at 8 weeks after transplantation. **b** Pie charts show the percentage of APC-positive and MBP-positive cells from grafted MSC-derived neural-like cells. **c** Grafted MSC-derived neural-like cells lost their affinity for the neuron markers (Tju-1-negative and microtubule-associated protein 2 (Map2)-negative) at 8 weeks after transplantation. At 3 days, the grafted MSC-derived neural-like cells were intensely immunostained with neuron markers (white arrowheads, Tju-1-positive and Map2-positive) and some of them began to express myelin-forming cell maker (white arrowhead, APC-positive). Scale bars = 25 μm. Tju-1, β-tubulin III

At 8 weeks after surgical procedure, survival and distribution of grafted GFP-positive cells in GS scaffold were clearly evident in most of the injury/graft sites of spinal cords. Thus, 53.58 % of grafted GFP-positive cells expressed the mature oligodendrocyte marker APC (Fig. 3a,b), and 31.12 % of the cells expressed the other mature oligodendrocyte marker MBP (Fig. 3a,b) in the injury/graft site in the MN + MT group. On the other hand, APC- and MBP-positive cells were not observed in the MSC group (Fig. 3a). Interestingly, Tju-1- and MAP2-positive cells were not observed in the MSC and MN + MT groups (Fig. 3c). To further explore whether differentiating MSCs could maintain the neural-like cell phenotype in the early period of transplantation, neural markers of the MSCs grafted were examined in the MN + MT group. The results showed that Tju-1-, Map2-, and APC-positive cells were observed in the injury/graft site of spinal cord in the MN + MT group 3 days after transplantation (Fig. 3c), suggesting that differentiating MSCs grafted can maintain the characteristic features of neural-like cells in the early period.

**Fig. 4:**
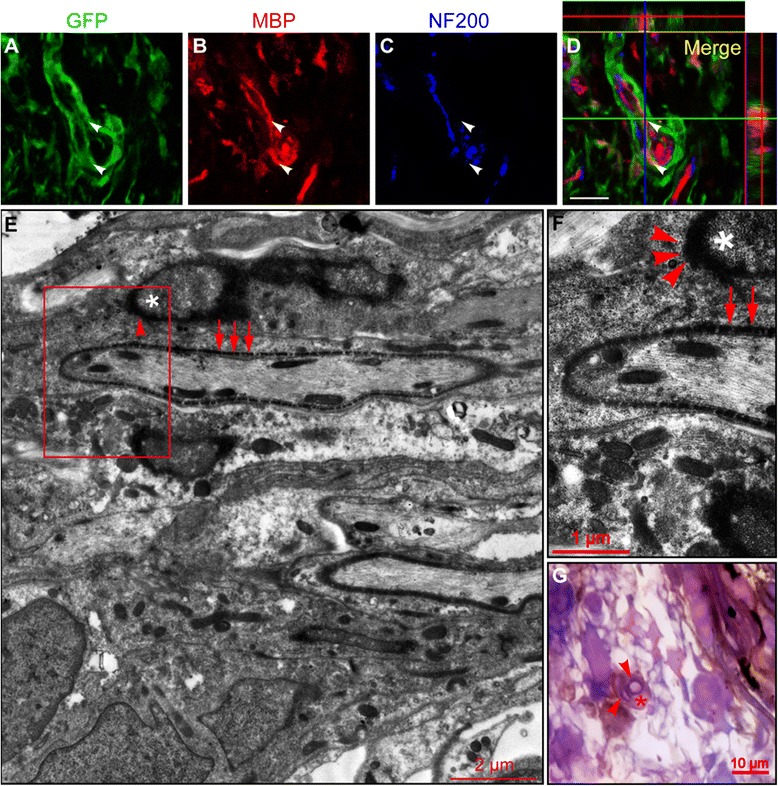
Myelin sheath formation in the injury/graft site of spinal cord at 8 weeks after mesenchymal stem cell (MSC)-derived neural-like cell transplantation. **a**–**d** Co-localization of green fluorescent protein (GFP) and myelin basic protein (MBP) in an MSC-derived neural-like cell as observed under a confocal microscope. The ortho section showed that the grafted GFP-positive cell was immunostained by MBP antibody and encircled the host neurofilament 200 (NF200)-positive axon (white arrowheads). **e**, **f** A myelin sheath was formed by a GFP-positive cell under the GFP-immunoelectron microscope observation. The red box in (**e**) was magnified in (**f**). **f** Red arrows indicated GFP-positive electron-dense 3, 3′-diaminobenzidine (DAB) deposited on the myelin sheath, and red arrowheads indicated GFP-positive electron-dense DAB deposited on the surface of nucleus. Asterisk indicates the nucleus of MSC-derived myelin-forming cell (**f**). **g** A GFP-positive cell deposited with DAB and counterstained by toluidine blue formed myelin-like structure (red arrowheads) in semithin section. Asterisk indicates DAB-positive cell body (**g**). Scale bars = 20 μm (a–d)

To investigate whether the APC and MBP expressing cells grafted could differentiate into mature oligodendrocytes, two techniques—namely, the triple IFS and GFP-immunoelectron microscope (GFP-IEM)—were used to detect myelination in the injury/graft site. Very strikingly, the tubular and ellipsoidal myelin-like structures, which were formed by the GFP-positive cells grafted, were observed by the triple IFS in the injury/graft site of spinal cord in all rats of the MN + MT group (Fig. 4a–d). Myelin-like structure showing coexistence appeared to encircle the host NF200-positive nerve fiber (Fig. 4a–d). To further verify that grafted MSCs could differentiate into myelinating cells, we scrutinized the GFP-positive cells by the GFP-IEM. The results showed that GFP-positive cells were identified in the injury/graft site and they appeared to enwrap a few host axons to form myelin structures. GFP reaction products of electron-dense DAB staining were obviously localized in the cytoplasm near or associated with the cell nucleus in a myelin-forming cell (Fig. 4e, f). Moreover, the myelin-like structures with DAB reaction products of GFP were found in the injury/graft site in the semi-thin section counterstained by toluidine blue (Fig. 4g). The results suggest that the differentiating MSCs grafted can transdifferentiate into myelin-forming cells in the injury/graft site of spinal cord transected completely at 8 weeks post-transplantation.

### Axonal regeneration and glial scar formation

**Fig. 5:**
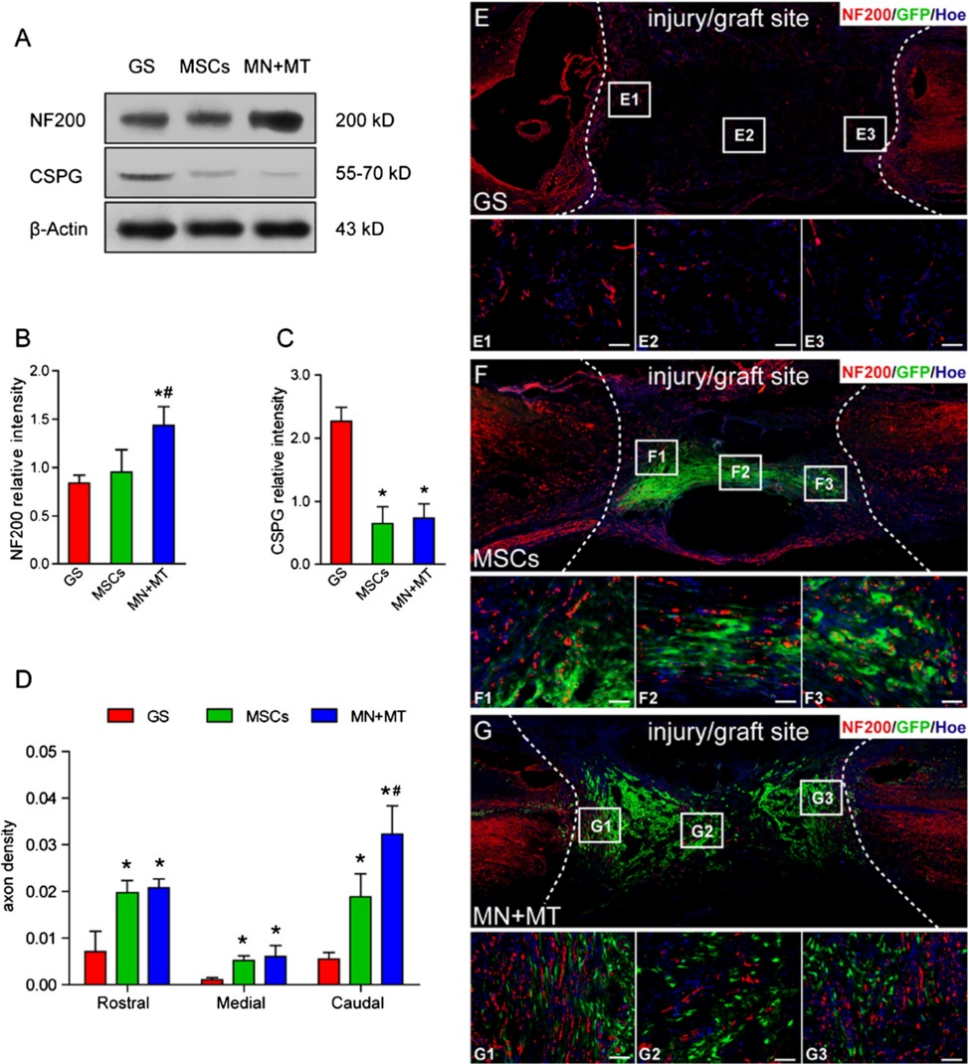
Assessment of axonal regeneration in the injury/graft site of spinal cord at 8 weeks after cell transplantation. **a** The expression of neurofilament 200 (NF200) and chondroitin sulfate proteoglycans clone CS-56 (CSPG) in the gelatin sponge (GS), mesenchymal stem cells (MSCs), and NT-3-MSCs (MN) + TrkC-MSCs (MT) groups was detected by Western blotting (WB). **b**, **c** Bar charts demonstrate semi-quantitative analysis of the level of NF200 and CSPG expressions. In the MN + MT group, the level of NF200 expression was higher than in the GS and MSCs groups (**b**) (*P < 0.05, ^*#*^ *P* < 0.05). The level of CSPG expression was lower in the MN + MT and MSCs groups compared with the GS group (**c**) (*P < 0.05). **d** Bar charts demonstrate the axon density in the rostral, central, and caudal areas of the injury/graft site. The axon density in the rostral and central areas of the injury/graft site was higher in the MN + MT and MSCs groups compared with the GS group (*P < 0.05). The axon density in the caudal area of the injury/graft site in the MN + MT group was higher than in the MSCs and GS groups (*P < 0.05, ^*#*^ *P* < 0.05). **e**–**g** NF200-positive nerve fiber regeneration in the GS, MSCs, and MN + MT groups. The enlarged images from the rostral, central, and caudal areas of the injury/graft site are shown in **E1–E3**, **F1–F3**, and **G1–G3**. One-way analysis of variance with least significant difference test statistics was performed to compare the axon density. Scale bars = 50 μm

At 8 weeks after surgery, 12 rats of the GS, MSC, and MN + MT groups (*n* = 4 for each group) were sacrificed for WB analysis to evaluate axonal regeneration and glial scar formation. The NF200 and CSPG expression level in the injury/graft site of spinal cord was detected in three groups (Fig. 5a). The NF200 expression level was higher, and CSPG expression was lower, in the MSC and MN + MT groups compared with the GS group (Fig. 5b, c; *P* < 0.05).

To assess the axonal growth in transected spinal cord at 8 weeks, NF200-positive fibers were examined with IFS in the rostral, central, and caudal areas of the injury/graft site (Fig. 5e–g). In the MSC and MN + MT groups, NF200-positive fibers were widely distributed at the injury/graft site (at the rostral, central, or caudal areas) when compared with the GS group (Fig. 5d; *P* < 0.05). However, in the rostral and central areas of the injury/graft site, the number of NF200-positive fibers between the MSC group and MN + MT group was not significantly different (Fig. 5d; *P* > 0.05). On the other hand, in the caudal area of the injury/graft site, the incidence of NF200-positive fibers in the MN + MT group was more than that in the MSC group (Fig. 5d; *P* < 0.05). The results indicate that genetically modified MSCs grafted may reduce the elements of glial scar formation and promote host axonal regeneration in the injury/graft site of spinal cord.

### Survival of injured host neurons

**Fig. 6:**
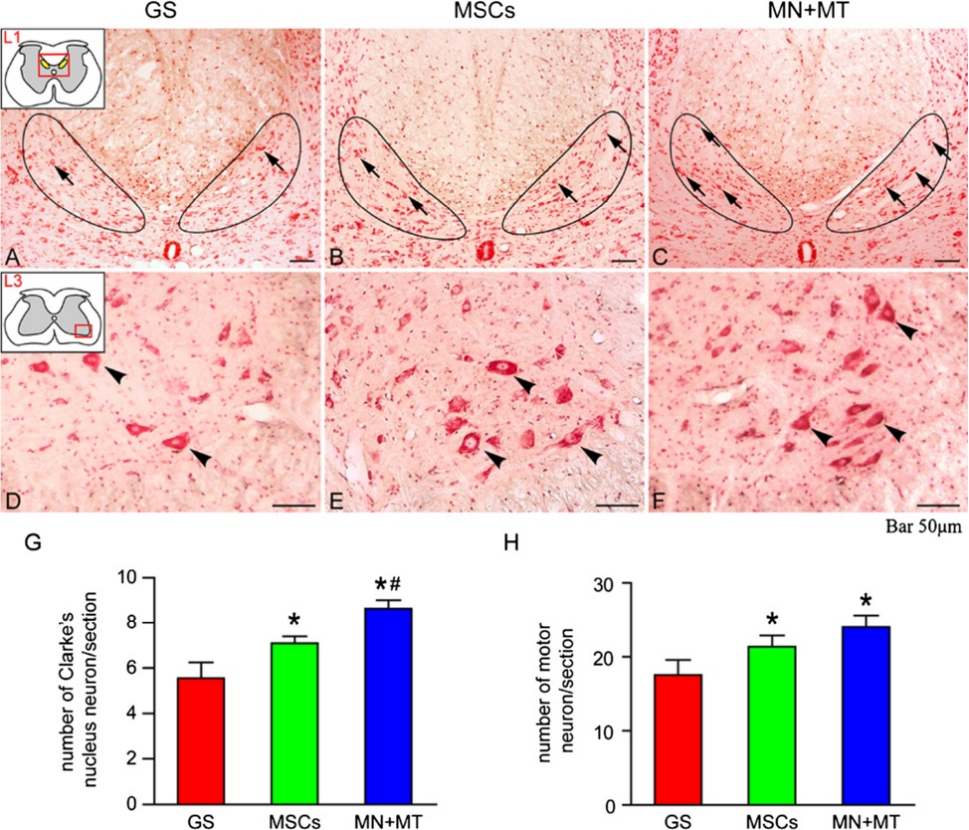
The survival of injured host neurons in L1 Clarke’s nucleus (CN) and L3 ventral horn of spinal cord at 8 weeks after cell transplantation. **a**–**c** Neural red staining of L1 CN in the gelatin sponge (GS) (**a**), mesenchymal stem cells (MSCs) (**b**), and NT-3-MSCs (MN) + TrkC-MSCs (MT) (**c**) groups. Arrows indicate the survival neurons in CN. **d**–**f** Neural red staining of L3 ventral horn in the GS (**d**), MSCs (**e**), and MN + MT (**f**) groups. Arrowheads indicate the survival neurons in ventral horn. **g, h** Bar charts show the number of survival neurons in CN and ventral horn. In the MN + MT group, the number of survival neurons in CN was more than in the MSCs and GS groups (**g**) (*P < 0.05, ^*#*^ *P* < 0.05). The number of survival neurons in ventral horn was more in the MSCs and MN + MT groups compared with the GS group (**h**) (*P < 0.05). One-way analysis of variance with least significant difference test statistics was performed to compare the number of survival neurons. Scale bars = 50 μm

To determine neuroprotection of the grafts in spinal cord transection, the survival of axotomized neurons in CN (L1 segment) and injured motor neurons (by transneuronal anterograde degeneration) in ventral horn (L3 segment) was evaluated with the neutral red staining (Fig. 6a–g). In the MN + MT group, the number of surviving CN neurons was more than in the GS and MSC groups (Fig. 6g; *P* < 0.05). In contrast to GS transplantation, MSC transplantation regimen also effectively rescued some of the axotomized host neurons (Fig. 6g). Furthermore, the number of survival of motor neurons in the MN + MT and MSC groups was significantly more than in the GS group (Fig. 6h). Overall, MN + MT transplantation treatment resulted in the highest number of surviving neurons axotomized (Fig. 6g; *P* < 0.05).

### Electrophysiological analysis and locomotor function

**Fig. 7:**
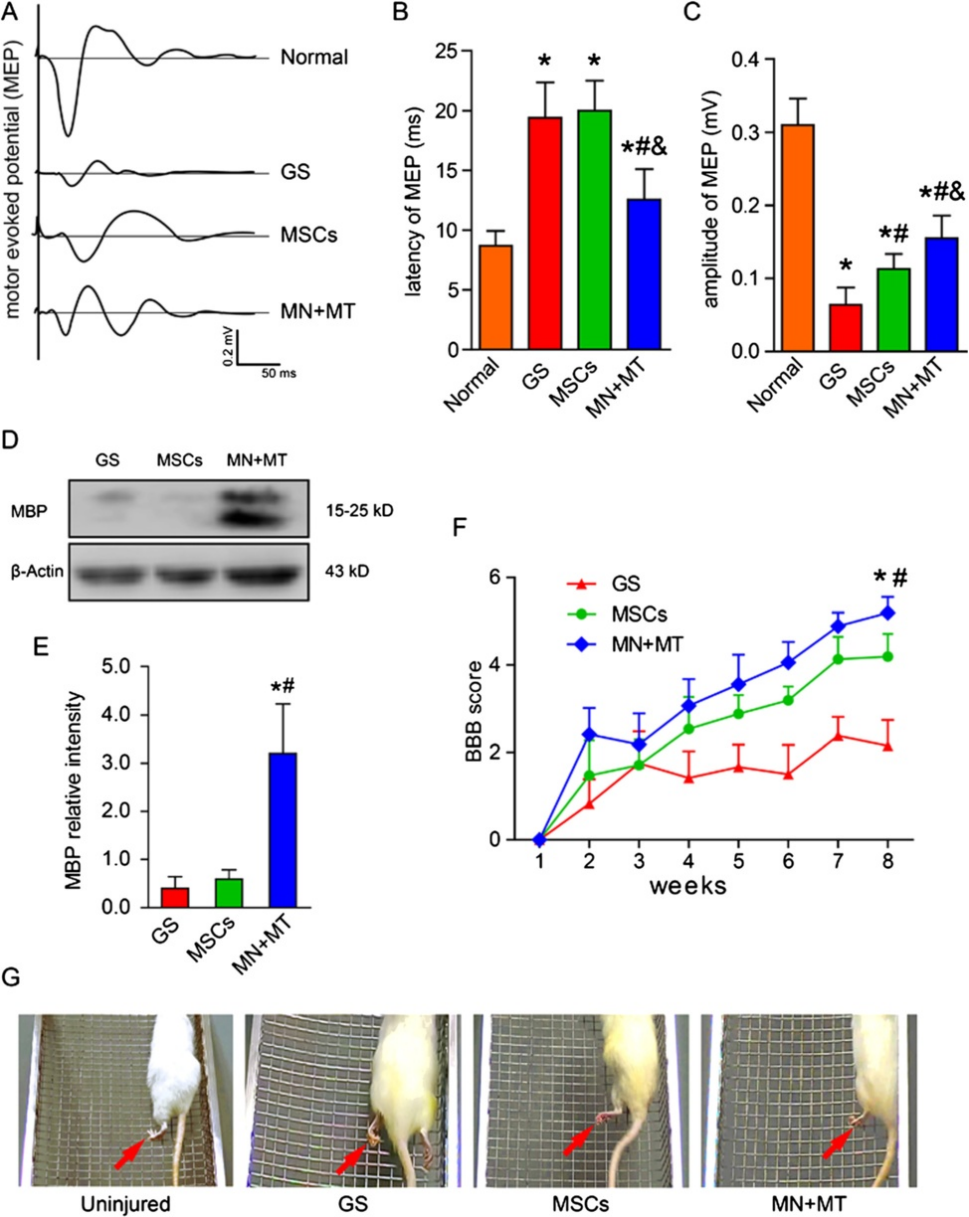
Outcomes of electrophysiology, myelin basic protein (MBP) protein level, and behavior at 8 weeks after cell transplantation. **a** Motor evoked potentials (MEPs) were obtained by electrophysiological analysis in the normal, gelatin sponge (GS), mesenchymal stem cells (MSCs), and NT-3-MSCs (MN) + TrkC-MSCs (MT) groups. **b,** **c** Bar charts of MEP latency and amplitude showed that shorter latency and higher amplitude of MEPs were exhibited in the MN + MT group compared with the GS (^*#*^ *P* < 0.05) and MSCs (^*&*^ *P* < 0.05) groups. **d** MBP expression in the GS, MSCs, and MN + MT groups was detected by Western blotting (WB). **e** Bar chart showed that the level of MBP expression was higher in the MN + MT group compared with the GS and MSCs groups (*P < 0.05, ^*#*^ *P* < 0.05). **f** Comparison of Basso, Beattie, and Bresnahan (BBB) score (mean ± standard deviation) of the handlimb locomotor function in spinal cord transected rats of the GS, MSCs, and MN + MT groups. In the MN + MT group, BBB score was higher than in the GS and MSCs groups (*P < 0.05, ^*#*^ *P* < 0.05). One-way analysis of variance with least significant difference test statistics was performed to compare the BBB score. **g** Three representative images of the handlimbs (red arrows) of the GS, MSCs, and MN + MT groups of rats which were climbing the inclined grids

As indicators of function and behavior recovery, electrophysiological measurement, BBB open-field locomotor rating scale, and grid climbing test were performed at 8 weeks post-transplantation. To confirm whether remyelination and axonal regeneration in the injury/graft site of spinal cord could be related with function recovery, we measured the MEPs in four groups of rats (Fig. 7a). In the normal group, stimulation at SMC evoked a large response amplitude (0.31 ± 0.04 mV) and shortened latency (8.70 ± 1.25 ms) of MEPs (Fig. 7b,c). Notably, in the MN + MT group, the response amplitude (0.16 ± 0.06 mV) and latency (12.55 ± 2.56 ms) of MEPs were significantly more improved (*P* < 0.05) than in the GS group (amplitude of 0.06 ± 0.02 mV and latency of 19.42 ± 2.95 ms) and the MSC group (amplitude of 0.11 ± 0.02 mV and latency of 20.00 ± 2.51 ms) (Fig. 7b,c). Through WB analysis, the level of MBP expression in the injury/graft site of spinal cord was the highest in the MN + MT group when compared with the GS and MSC groups (Fig. 7d, e; *P* < 0.05). The results suggest that remyelination in the injury/graft site is increased in the MN + MT group, which may be linked to the nerve conduction improvement.

The hindlimbs of the other three groups of rats, except for the normal group, were completely paralyzed after their T10 spinal cord segment was transected. At the first week after SCI, the hindlimb locomotion was impaired severely and no difference in BBB scores was observed in any group (Fig. 7f). Locomotion performance was gradually improved in two groups with cell transplantation starting 2 weeks after SCI, and the mean score of the MN + MT group was significantly higher (*P* < 0.05) than those of other groups (Fig. 7f), indicating that rats of the MN + MT group are able to move three joints of the hindlimbs. The 45° inclined grid climbing was also performed to evaluate spontaneous placing reflex triggered by direct contact at the eighth week after SCI (Fig. 7g). The result showed that, before surgery, the body of the uninjured rats could be supported by their hindlimbs and that the rats exhibited the coordination movement of fore-hindlimbs (Fig. 7g; Additional file 1). In the GS group, rats had no placing reflex when climbing the inclined grid with their forelimbs, and their hindlimbs simply dragged along (Fig. 7g; Additional file 2). In the MSC group, the rats stepped on the grid with the back of their paws and exhibited spasmodic movement occasionally (Fig. 7g; Additional file 3). However, in the MN + MT group, the rats exhibited the strongest hindlimb placing reflex, stepping on the rung with the plantar surface of the paws; some rats exhibited coordination movement of the fore-hindlimbs, and fewer footfalls were made when stepping on the grid (Fig. 7g; Additional file 4).

## Discussion

Our previous studies indicate that NT-3 overexpressing SCs can promote MSCs or neural stem cells overexpressing TrkC to differentiate into neuron-like cells in vitro or in injured spinal cord [22, 27, 46]. The findings revealed a key role of NT-3/TrkC signal transduction in differentiation of neuron-like cells. It is well documented that SCs secrete various NTFs. To obviate the undesirable effects of these factors, MSCs were opted for instead as the NT-3 delivery vehicle in this study to demonstrate the important role of NT-3/TrkC pathway on neural differentiation of MSCs. The present results suggest that NT-3/TrkC interaction can promote differentiation of MSCs into neural-like cells, such as neuron-like cells and astrocyte-like cells, in the 3D GS scaffold in vitro. Interestingly, some differentiating MSCs appeared to lose their acquired neural features and transdifferentiated into myelinating cells when they were transplanted into the injury gap of spinal cord transected completely 8 weeks later. This suggests that the complex niche of the injury/graft site may play an important role in regulating the transdifferentiation of the MSCs. Furthermore, the grafted cells partially improved the locomotion of paralyzed hindlimbs as well as the outcome of functional electrophysiological measurement that was accompanied by axonal regeneration and remyelination.

TrkC, known as a high-affinity catalytic receptor of NT-3, mediates multiple effects during neural development, such as survival and differentiation of neurons [47, 48]. We showed here that genetically modified MSCs differentiated into neural-like cells expressing the neuron markers (Tju-1 and Map2) and synaptic components (PSD95 and SYP) rather than astrocyte-like cells after the TrkC-MSCs co-cultured with the NT-3-MSCs in a 3D GS scaffold at 14 days in vitro. In general, expression of neuron markers increased while that of astrocyte (namely, GFAP) gradually diminished with increased culture time. It was estimated that less than 10 % cells expressed GFAP at 3 and 7 days after culture (Additional file 5: Figure S1). It is noteworthy that the percentage of MAP2-positive cells in the MN + MT group was significantly higher than in the MN and the MT groups, indicating the important role of NT-3/TrkC interaction in neuron-like cell differentiation of MSCs. The underlying molecular pathways regulating this, however, are uncertain. Jori et al. reported that MEK-ERK signaling could contribute to neural commitment and differentiation of MSCs [49]. It has also been reported that NT-3 stimulation of TrkC upregulated proneuronal genes promoting neuronal differentiation through activating the Erk1/2 MAP kinase pathway in the immature homogeneous subpopulation of human MSCs and activated the Arf6-Rac1 pathway upregulating proneuronal genes in HEK293 cells [25, 50].

Although some neural features have been demonstrated in vitro in the differentiating MSCs*,* it remains to be ascertained whether these could be retained in vivo. In this study, we transplanted differentiating MSCs in a scaffold (the MN + MT group) into the transected site of the spinal cord. It is striking to note that, at 8 weeks after grafting, the differentiating MSCs were negative for Tju-1 and MAP2 IFS at 3 days. Likewise, GFAP staining was negative (data not shown). A striking feature was the occurrence of some myelin-like structures formed by some of the differentiating MSCs grafted with MBP and APC IFS. This corroborates our previous studies which showed that MSCs verexpressing TrkC by pre-induced with NT-3 and retinoic acid could differentiate into oligodendrocyte-like cells in different spinal cord disease models [15, 51]. It remains unclear why only some differentiating MSCs transdifferentiate to become myelin-forming cells in the microenvironment of SCI. It has been reported that MSC-derived 5-hydroxytryptamine-sensitive neurons could dedifferentiate and redifferentiate under different conditions [52]. Furthermore, adult rat MSCs can be switched from neuronal phenotypic cells to epithelial phenotypic cells or vice versa. It was suggested that the plasticity of MSCs depends on their ability to dedifferentiate into the more primitive state [53]. Here, we have analyzed the mRNA transcription of the pluripotency markers *Sox2*, *Nanog*, *Oct4*, and *Alp* in differentiating MSCs of the MN + MT group in vitro at the 14th day by quantitative real-time polymerase chain reaction (Additional file 6: Figure S2A). The expression of all of the mRNAs, except for *Alp* mRNA, was significantly higher in the MN + MT group compared with the M group. The transcription of pluripotency genes indicates the flexible characteristics of MSC-derived neural-like cells. The IFS also showed that some differentiated MSCs in the MN + MT group could still express Nestin, Sox2, Oct4, and Nanog proteins (Additional file 7: Figure S3A–D). However, these MSCs lost the capacity of pluripotency protein expression at 8 weeks after being transplanted into the injury site of spinal cord (Additional file 7: Figure S3E–H), and this may be related to transplanted MSCs transdifferentiated into mature myelin-forming cells. In light of the above, we consider that MSC-derived neural-like cells might dedifferentiate into more primitive stem cells, which are reprogrammed into myelinating cells in response to the complex milieu of the injury/graft site of spinal cord transected. This may be a possible explanation for MSC-derived neural-like cells transdifferentiating into the myelin-forming cells in vivo in our study.

After spinal cord transection, local tissue destruction and necrosis promote formation of a permanent gap and glia scar at the injury site. How to effectively bridge this separated rostral and caudal stumps and how to reorganize the local neural circuits have become priorities for spinal cord repair [54]. Our strategy is transplanting a 3D GS scaffold containing genetically modified MSCs to construct a potential and functional neural circuit by trophic support and immunomodulation. In this study, we found that the number of surviving neurons in L1 CN and L3 ventral horn was significantly improved after cell transplantation. NF200-positive axons extending into the injury/graft site were significantly increased in numbers, especially after co-transplanting NT-3-MSCs + TrkC-MSCs. Such effects may be facilitated by partial restoration of target-derived trophic support (e.g., NT-3 by NT-3-MSCs) to L1 CN neurons and L3 ventral horn neurons which were undergoing retrograde degeneration or anterograde transneuronal degeneration after the T10 spinal cord segment was transected [55]. In addition, we have found that the differentiating MSCs maintained some immunomodulatory characteristics in vitro at the 14th day, such as mRNA transcription of prostaglandin E synthase (*Ptges*), nitric oxide synthase 2 (*Nos2*), transforming growth factor beta (*Tgfb*), and leukemia inhibitory factor (*Lif*) (Additional file 6: Figure S2B). Transcription of these mRNAs may inhibit T-cell proliferation and natural killer cell function, relieve graft-versus-host reaction, and promote the survival of transplanted cells [56, 57]. They may also play an important role in nerve regeneration and glia scar reducing, a notion that would be in line with our WB results that the grafted cells significantly increased the level of NF200 and reduced CSPG synthesis in the injury/graft site.

Interestingly, in the MN + MT group, there was no significant difference in mRNA transcription of immunomodulatory factors, except for *Lif* mRNA, compared with the M group. *Lif* mRNA was more than threefold higher in the MN + MT group than in the M group, indicating that *Lif* may play some special roles at the injury site. *Lif*, as an interleukin 6 class cytokine, plays an important role in both the immune and nervous systems [58, 59]. Taha et al. reported that Lif could upregulate pluripotency markers *Oct4*, *Nanog*, and *Sox2* mRNAs in adipose tissue-derived stem cells [60]. This is consistent with our results that transcription of *Nanog* and *Oct4* mRNAs was higher in the MN + MT group. Additionally, Lif can promote oligodendrocyte precursors to survive and differentiate as well as remyelination [61] and also can induce some stem cells differentiating into myelin-forming cells [62]. It is therefore speculated that Lif not only can act as an immune modulator but also can maintain the “stemness” of MSC-derived neural-like cells by inducing them to transdifferentiate into myelin-forming cells in the injury/graft site of spinal cord.

In the present study, all rats receiving cell transplantation displayed moderate open-field locomotion and inclined grid climbing improvement. In these, NF200-positive axons appeared to extend into the injury/graft site of spinal cord with some of the regenerating axons being myelinated. On closer analysis, however, it was found that rats receiving NT-3-MSC + TrkC-MSC co-transplantation demonstrated the most extensive hindlimb movement without body weight-supporting stepping and limited coordination movement of fore-hindlimbs. The possibility is therefore considered that this was attributable to regrowth of some supraspinal input axons into the injury/graft site through the entire lesion area to activate central pattern generator for hindlimb locomotion [63]. Although our results show that the hindlimb “placing reflex” and coordination movement of fore-hindlimbs were initiated by climbing the inclined grid after NT-3-MSC + TrkC-MSC co-transplantation treatment, it does not necessarily mean that there is enough “message relay” between the cerebral cortex and caudal area of the injury/graft site of the spinal cord. This is because climbing the inclined grid can be triggered without being directly initiated by the motor cortex in the SCI rats [45]. The improvement of electrophysiological properties, as an important treatment effect evaluation, was manifested by a higher amplitude and shorter latency of MEP in the MN + MT group, which may be beneficial to reorganization and myelination of the axotomized nerve fibers.

The present results indicate that genetically modified MSCs can transdifferentiate into myelin-forming cells in the complex niches after their transplantation in the injured spinal cord. Thus, NT-3 overexpressing MSCs retained immunomodulatory effects that may be beneficial to regeneration of axons, survival of host injured neurons, and improvement of locomotion. On the other hand, in view of the limited functional improvement, it seems obvious that monotherapy for severe SCI is inadequate. Therefore, polytherapy should be explored as a strategy for complex neurological maladies [54]. Further studies focusing on the underlying mechanism guiding the transdifferentiation in vivo in genetically modified MSCs would therefore be desirable.

## Conclusions

Using adenovirus as a vector to transfect MSCs with NT-3 gene and its receptor TrkC gene, respectively, the present study constructs an implant of 3D GS scaffold with the MSCs to restore the spinal cord organization after T10 segment transection. The MSCs modified genetically could differentiate into neural-like cells and maintain some immunomodulatory characteristics in the 3D GS scaffold in vitro. After the MSC-derived neural-like cells were transplanted into the injury site of rat spinal cord, they appeared to lose some neural phenotypes, and remarkably some of them could transdifferentiate into myelin-forming cells at 8 weeks. MSC-derived myelin-forming cells established myelin sheaths with the host regenerating axons. The results suggest that the complex milieu of the injury/graft site of spinal cord can be improved by grafted MSCs modified genetically to protect the host injured neurons and promote their axon regeneration. Simultaneously, the microenvironment of the injury/graft site can induce differentiating MSCs modified genetically to transdifferentiate for restoring the structure and function of injured spinal cord.

## Additional files

Additional file 1:
**Before surgery, the body of the uninjured rats could be supported by their hindlimbs and the rats exhibited the coordination movement of fore-hindlimbs.**
Additional file 2:
**In the gelatin sponge (GS) group, rats had no placing reflex when climbing the inclined grid with their forelimbs, and their hindlimbs simply dragged along.**
Additional file 3:
**In the mesenchymal stem cell (MSC) group, the rats stepped on grid with the backs of their paws and exhibited the spasmodic movement occasionally.**
Additional file 4:
**In the NT-3-MSC (MN) + TrkC-MSC (MT) group, the rats exhibited the strongest hindlimb placing reflex, stepping on the rung with the plantar surface of the paws; some rats exhibited the coordination movement of the fore-hindlimbs, and fewer footfalls were made when stepping on the grid.**
Additional file 5: Figure S1. Neural differentiation of co-cultured TrkC-MSCs and NT-3-MSCs in the 3D GS scaffold at 3 and 7 days after culture. **a** IFS shows the distribution of Tju-1-positive, Map2-positive, and GFAP-positive cells in the M, MN, MT, and MN + MT groups. Arrows indicate three kinds of positive cells. **b-d** Bar charts show the percentage of Tju-1-positive, Map2-positive, and GFAP-positive cells in all groups. Tju-1-positive cells are significantly increased in the MN + MT groups compared with the M, MN, and MT groups at 3 days but not at 7 days **(b)** (**P* < 0.05, ^#^ *P* < 0.05, &*P* < 0.05). At 3 days, Map2-positive cells are absent in all groups. However, at 7 days, Map2-positive cells are detected. When compared with the M group, genetically modified MSCs exhibit a higher incidence of Map2-positive cells **(c)** (**P* < 0.05). In all groups, the percentage of GFAP-positive cells is about 10 % and there is no significant difference among them **(d)** (*P* > 0.05). Asterisks indicate statistical significance compared with the M group (**P* < 0.05), pound sign indicates significance compared with the MN group (^#^ *P* < 0.05), and ampersand indicates significance compared with the MT group (&*P* < 0.05). One-way analysis of variance with least significant difference test statistics was performed to compare the percentage of positive cells. Scale bars = 20 μm. 3D, Three-dimensional; GFAP, Glial fibrillary acidic protein; GS, Gelatin sponge; IFS, Immunofluorescence staining; M, MSC; Map2, Microtubule-associated protein 2; MN, NT-3-MSCs; MSC, Mesenchymal stem cell; MT, TrkC-MSCs; NT-3, Neurotrophin-3; Tju-1, β-tubulin III; TrkC, Tropomyosin receptor kinase C. Additional file 6: Figure S2. For quantitative real-time polymerase chain reaction (qRT-PCR) analysis in Methods, total mRNAs of the mesenchymal stem cells (MSCs) were extracted by using TRIZOL reagent (Invitrogen, a brand of Thermo Fisher Scientific, Waltham, MA, USA) from the MSC (M) (n = 3) and NT-3-MSC (MN) + TrkC-MSC (MT) (n = 3) groups at 14 days after culture in vitro. After this, the cDNAs were synthesized by using cDNA Cycle Kit (Takara Bio Inc., Otsu, Japan). One microlitre of reverse-transcription product of each cDNA was amplified with gene-specific primers and the SYBRGreen PCR Master mix (TaKaRa Bio Inc.) by Bio-Rad iCycler iQ5 PCR (Bio-Rad Laboratories, Hercules, CA, USA). PCR conditions were performed by initial denaturation at 95 °C for 2 min and 40 cycles with 95 °C for 5 s and 60 °C for 30 s. The primer sequences (forward, reverse) were as follows: Sox2 (forward: 5′-GAACTAGACTCGGGCGATG-3′, reverse: 5′-CCCAGCAAGAACCCTTTCT-3′); Nanog (forward: 5′-TGACTCAGAAGGGCTCAGCG-3′, reverse: 5′-TATGGAGCGGAG-CAGCGT-3′); Oct4 (forward: 5′-GCCAGGCAGGAGCACGAG-3′, reverse: 5′-GCTTTCATATCCTGGGACTCCT-3′); Alp (forward: 5′-ACAG- AACTGATGTGGAATATGAAC-3′, reverse: 5′-GGGAGTGCTTGTGTC- TAGGTT-3′); prostaglandin E synthase (Ptges) (forward: 5′-GCTGCGGAAGAAGGCTTTTG-3′, reverse: 5′-CCAGGAATGAGTAGACGAAACCAA-3′); nitric oxide synthase 2 (Nos2) (forward: 5′-AGAGTGAGAAGTCCAGCCGC-3′, reverse: 5′-GAAGAACAATCCACAACTCGC-3′); transforming growth factor beta (Tgfb) (forward: 5′-TGCTTTAGGAATGTGCAGGATAA-3′, reverse: 5′-GCTTCGGGGTTTATGGTGTT-3′); leukemia inhibitory factor (Lif) (forward: 5′-TTTCCTATTACACAGCTCAAGGGG-3′, reverse: 5′-TTGTTGCACAGACGGCAAAG-3′); and β-actin (forward: 5′-AGAGGGAAATCGTGCGTGAC-3′, reverse: 5′-AGAGGTCTTTACGGATGTCAACG-3′). The results of qRT-PCR analysis showed that **(a)** the level of mRNA transcription of the pluripotency markers Sox2, Nanog, and Oct4 in the MN + MT group at 14 days after culture was higher than that in the M group (**P* < 0.05), except for the pluripotency marker Alp mRNA (*P* > 0.05), and **(b)** the level of mRNA transcription of immunomodulatory factors Ptges, Nos2, Tgfb, and Lif is not significantly different between the M and MN + MT groups (*P* > 0.05), except for the immunomodulatory factor Lif mRNA level that is higher in the MN + MT group (**P* < 0.05). Additional file 7: Figure S3. Expression of pluripotency markers in differentiated mesenchymal stem cells (MSCs) before and after their transplantation. **a-d** Expression of pluripotency markers Nestin, Sox2, Oct4, and Nanog was detected in the MSCs of the NT-3-MSC (MN) + TrkC-MSC (MT) group after genetically modified MSCs were cultured in three-dimensional gelatin sponge (3D GS) scaffold for 14 days. **e-h** Differentiated MSCs lost their pluripotency markers after being transplanted into injured spinal cord at 8 weeks. Scale bars = 20 μm in (a-d) and 40 μm in (e, f).

## Abbreviations

2D: Two-dimensional
3D: Three-dimensional
APC: Adenomatous polyposis coli
BBB: Basso, Beattie, and Bresnahan
BCA: Bicinchoninic acid assay
CN: Clarke’s nucleus
CSPG: Chondroitin sulfate proteoglycans clone CS-56
DAB: 3, 3′-Diaminobenzidine
DMEM: Dulbecco’s modified Eagle’s medium
FBS: Fetal bovine serum
GFAP: Glial fibrillary acidic protein
GFP: Green fluorescent protein
GS: Gelatin sponge
HRP: Horseradish peroxidase
IEM: Immune-electron microscope
IFS: Immunofluorescence staining
Lif: Leukemia inhibitory factor
Map2: Microtubule-associated protein 2
MBP: Myelin basic protein
MEP: Motor evoked potential
MOI: Multiplicity of infection
MN: NT-3-MSCs
MSC: Mesenchymal stem cell
MT: TrkC-MSCs
NF200: neurofilament 200
NT-3: Neurotrophin-3
NTF: Neurotrophic factor
PB: Phosphate buffer
PBS: Phosphate-buffered saline
SC: Schwan cell
SCI: Spinal cord injury
SD: Sprague-Dawley
SEM: Scanning electron microscope
SMC: Sensorimotor cortex
TEM: Transmission electron microscope
Tju-1: β-tubulin III
TrkC: Tropomyosin receptor kinase C
WB: Western blotting
