# Supplementary material for: Donor mesenchymal stem cell-derived neural-like cells transdifferentiate into myelin-forming cells and promote axon regeneration in rat spinal cord transection
Source: Stem Cell Res Ther. 2015 May 27;6(1):105. doi: 10.1186/s13287-015-0100-7 (PMC4482203; doi:10.1186/s13287-015-0100-7)
Supplement: Additional file 5: Figure S1. — Neural differentiation of co-cultured TrkC-MSCs and NT-3-MSCs in the 3D GS scaffold at 3 and 7 days after culture. a IFS shows the distribution of Tju-1-positive, Map2-positive, and GFAP-positive cells in the M, MN, MT, and MN + MT groups. Arrows indicate three kinds of positive cells. b-d Bar charts show the percentage of Tju-1-positive, Map2-positive, and GFAP-positive cells in all groups. Tju-1-positive cells are significantly increased in the MN + MT groups compared with the M, MN, and MT groups at 3 days but not at 7 days (b) (*P < 0.05, # P < 0.05, &P < 0.05). At 3 days, Map2-positive cells are absent in all groups. However, at 7 days, Map2-positive cells are detected. When compared with the M group, genetically modified MSCs exhibit a higher incidence of Map2-positive cells (c) (*P < 0.05). In all groups, the percentage of GFAP-positive cells is about 10 % and there is no significant difference among them (d) (P > 0.05). Asterisks indicate statistical significance compared with the M group (*P < 0.05), pound sign indicates significance compared with the MN group (# P < 0.05), and ampersand indicates significance compared with the MT group (&P < 0.05). One-way analysis of variance with least significant difference test statistics was performed to compare the percentage of positive cells. Scale bars = 20 μm. 3D, Three-dimensional; GFAP, Glial fibrillary acidic protein; GS, Gelatin sponge; IFS, Immunofluorescence staining; M, MSC; Map2, Microtubule-associated protein 2; MN, NT-3-MSCs; MSC, Mesenchymal stem cell; MT, TrkC-MSCs; NT-3, Neurotrophin-3; Tju-1, β-tubulin III; TrkC, Tropomyosin receptor kinase C. [file 13287_2015_100_MOESM5_ESM.doc]

**Additional figure** **1.** Neural differentiation of co-cultured TrkC-MSCs and NT-3-MSCs in the 3D GS scaffold at 3 and 7 days after culture. (A) IFS shows the distribution of Tju-1-positive, Map2-positive and GFAP-positive cells in the M, MN, MT and MN+MT groups. Arrows indicate three kinds of positive cells. (B-D) Bar charts show the percentage of Tju-1-positive, Map2-positive and GFAP positive cells in all groups. Tju-1-positive cells are significantly increased in the MN+MT groups compared with the M, MN and MT groups at 3 days but not at 7 days (B, **P* < 0.05, ^#^*P* < 0.05, ^&^*P* < 0.05). At 3 days, Map2-positive cells are absent in all groups. However, at 7 days, Map2-positive cells are detected. When compared with the M group, genetically modified MSCs exhibit a higher incidence of Map2-positive cells (C, **P* < 0.05). In all groups, the percentage of GFAP-positive cells is about 10% and there is no significant difference among them (D, *P* > 0.05). Asterisks indicate statistical significance compared with the M group (**P* < 0.05), pound indicates significance compared with the MN group (^#^*P* < 0.05), and ampersand indicates significance compared with the MT group (^&^*P* < 0.05). One-way ANOVA with LSD test statistics was performed to compare the percentage of positive cells. Scale bars = 20 μm.
