# Supplementary material for: Donor mesenchymal stem cell-derived neural-like cells transdifferentiate into myelin-forming cells and promote axon regeneration in rat spinal cord transection
Source: Stem Cell Res Ther. 2015 May 27;6(1):105. doi: 10.1186/s13287-015-0100-7 (PMC4482203; doi:10.1186/s13287-015-0100-7)
Supplement: Additional file 6: Figure S2. — For quantitative real-time polymerase chain reaction (qRT-PCR) analysis in Methods, total mRNAs of the mesenchymal stem cells (MSCs) were extracted by using TRIZOL reagent (Invitrogen, a brand of Thermo Fisher Scientific, Waltham, MA, USA) from the MSC (M) (n = 3) and NT-3-MSC (MN) + TrkC-MSC (MT) (n = 3) groups at 14 days after culture in vitro. After this, the cDNAs were synthesized by using cDNA Cycle Kit (Takara Bio Inc., Otsu, Japan). One microlitre of reverse-transcription product of each cDNA was amplified with gene-specific primers and the SYBRGreen PCR Master mix (TaKaRa Bio Inc.) by Bio-Rad iCycler iQ5 PCR (Bio-Rad Laboratories, Hercules, CA, USA). PCR conditions were performed by initial denaturation at 95 °C for 2 min and 40 cycles with 95 °C for 5 s and 60 °C for 30 s. The primer sequences (forward, reverse) were as follows: Sox2 (forward: 5′-GAACTAGACTCGGGCGATG-3′, reverse: 5′-CCCAGCAAGAACCCTTTCT-3′); Nanog (forward: 5′-TGACTCAGAAGGGCTCAGCG-3′, reverse: 5′-TATGGAGCGGAG-CAGCGT-3′); Oct4 (forward: 5′-GCCAGGCAGGAGCACGAG-3′, reverse: 5′-GCTTTCATATCCTGGGACTCCT-3′); Alp (forward: 5′-ACAG- AACTGATGTGGAATATGAAC-3′, reverse: 5′-GGGAGTGCTTGTGTC- TAGGTT-3′); prostaglandin E synthase (Ptges) (forward: 5′-GCTGCGGAAGAAGGCTTTTG-3′, reverse: 5′-CCAGGAATGAGTAGACGAAACCAA-3′); nitric oxide synthase 2 (Nos2) (forward: 5′-AGAGTGAGAAGTCCAGCCGC-3′, reverse: 5′-GAAGAACAATCCACAACTCGC-3′); transforming growth factor beta (Tgfb) (forward: 5′-TGCTTTAGGAATGTGCAGGATAA-3′, reverse: 5′-GCTTCGGGGTTTATGGTGTT-3′); leukemia inhibitory factor (Lif) (forward: 5′-TTTCCTATTACACAGCTCAAGGGG-3′, reverse: 5′-TTGTTGCACAGACGGCAAAG-3′); and β-actin (forward: 5′-AGAGGGAAATCGTGCGTGAC-3′, reverse: 5′-AGAGGTCTTTACGGATGTCAACG-3′). The results of qRT-PCR analysis showed that (a) the level of mRNA transcription of the pluripotency markers Sox2, Nanog, and Oct4 in the MN + MT group at 14 days after culture was higher than that in the M group (*P < 0.05), except for the pluripotency marker Alp mRNA (P > 0.0 [file 13287_2015_100_MOESM6_ESM.doc]

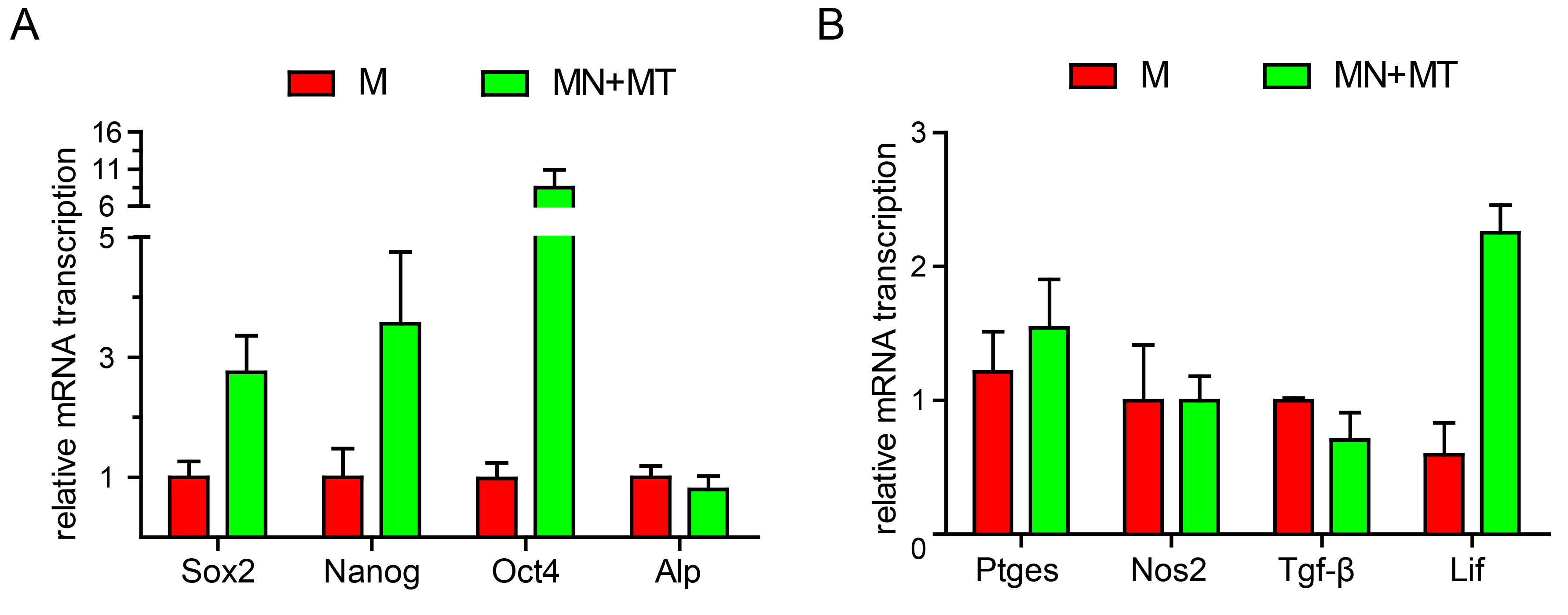


**Additional figure 2.** For quantitative real-time polymerase chain reaction (qRT-PCR) analysis, in materials and methods, total mRNAs of the MSCs were extracted using TRIZOL reagent (Invitrogen, USA) from the M (*n* = 3) and MN+MT (*n* = 3) groups at 14 days after culture *in vitro*. And then, the cDNAs were synthesized using cDNA Cycle Kit (TaKaRa Bio Inc., Otsu, Japan). One μl reverse-transcription product of each cDNA was amplifiered with gene-specific primers and the SYBRGreen PCR Master mix (TaKaRa Bio Inc.,Otsu, Japan) by Bio-Rad iCycler iQ5 PCR (BioRad, USA). PCR conditions were performed by initial denaturation at 95°C for 2 min and 40 cycles with 95°C for 5 s, 60°C for 30 s. The primer sequences (forward, reverse) were as follows: Sox2 (forward: 5'-GAACTAGACTCGGGCGATG-3’, reverse: 5’-CCCAGCAAGAACCCTTTCT-3’); Nanog (forward: 5’-TGACTCAGAAGGGCTCAGCG-3’, reverse: 5’-TATGGAGCGGAG-CAGCGT-3’); Oct4 (forward: 5'-GCCAGGCAGGAGCACGAG-3’, reverse: 5’-GCTTTCATATCCTGGGACTCCT-3’); Alp (forward: 5’-ACAG- AACTGATGTGGAATATGAAC-3’, reverse: 5’-GGGAGTGCTTGTGTC- TAGGTT-3’); prostaglandin E synthase (Ptges, forward: 5’- GCTGCGGAAGAAGGCTTTTG-3’, reverse: 5’-CCAGGAATGAGTAGACGAAACCAA-3’); Nitric oxide synthase 2 (Nos2, forward: 5’-AGAGTGAGAAGTCCAGCCGC-3’, reverse: 5’-GAAGAACAATCCACAACTCGC-3’); Transforming growth factor beta (Tgfb, forward: 5’-TGCTTTAGGAATGTGCAGGATAA-3’, reverse: 5’-GCTTCGGGGTTTATGGTGTT-3’); Leukemia inhibitory factor (Lif, forward: 5’-TTTCCTATTACACAGCTCAAGGGG-3’, reverse: 5’-TTGTTGCACAGACGGCAAAG-3’); β-actin (forward: 5’- AGAGGGAAATCGTGCGTGAC-3’, reverse: 5’- AGAGGTCTTTACGGATGTCAACG -3’). The results of qRT-PCR analysis showed (A) the level of mRNAs transcription of the pluripotency markers Sox2, Nanog and Oct4 in the MN+MT group at 14 days after culture was higher than that in the M group (*P < 0.05), except for the pluripotency marker Alp mRNA (P > 0.05). (B) The level of mRNAs transcription of immunomodulatory factors Ptges, Nos2, Tgfb and Lif is not significantly different between the M and MN+MT groups (P > 0.05), except for the immunomodulatory factor Lif mRNA level that is higher in the MN+MT group (*P < 0.05).
