# Supplementary material for: Donor mesenchymal stem cell-derived neural-like cells transdifferentiate into myelin-forming cells and promote axon regeneration in rat spinal cord transection
Source: Stem Cell Res Ther. 2015 May 27;6(1):105. doi: 10.1186/s13287-015-0100-7 (PMC4482203; doi:10.1186/s13287-015-0100-7)
Supplement: Additional file 7: Figure S3. — Expression of pluripotency markers in differentiated mesenchymal stem cells (MSCs) before and after their transplantation. a-d Expression of pluripotency markers Nestin, Sox2, Oct4, and Nanog was detected in the MSCs of the NT-3-MSC (MN) + TrkC-MSC (MT) group after genetically modified MSCs were cultured in three-dimensional gelatin sponge (3D GS) scaffold for 14 days. e-h Differentiated MSCs lost their pluripotency markers after being transplanted into injured spinal cord at 8 weeks. Scale bars = 20 μm in (a-d) and 40 μm in (e, f). [file 13287_2015_100_MOESM7_ESM.docx]

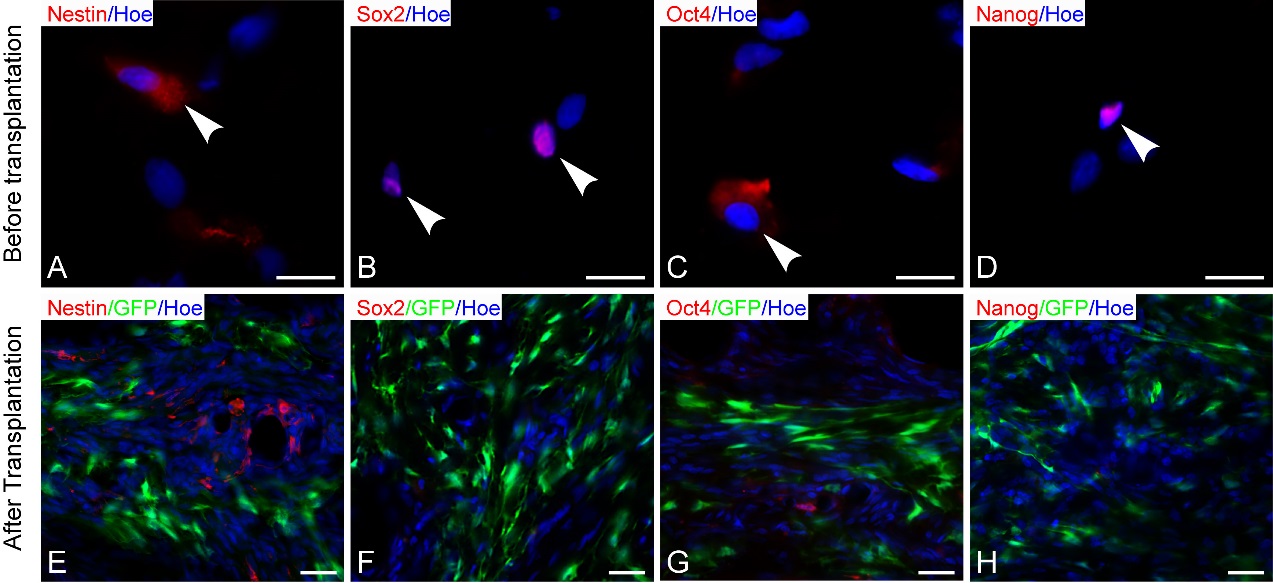


**Additional figure 3.** The pluripotency marker expression was displayed before or

after differentiated MSCs were transplanted. **(A**-**D)** Show that expression of the

pluripotency markers, namely, Nestin, Sox2, Oct4 and Nanog expression in the

MSCs of the MN+MT group after genetically modified MSCs were cultured in

3D GS scaffold for 14 days. **(E**-**H)** Differentiated MSCs lost the expression of

pluripotency markers after they being transplanted into injured spinal cord at 8

weeks. Scale bars = 20 μm in **(A**-**D)**, 40 μm in **(E**-**F)**.
